# Replicating RNA vaccine confers durable immunity against Crimean Congo hemorrhagic fever virus challenge in mice

**DOI:** 10.1038/s41541-024-01045-1

**Published:** 2024-12-19

**Authors:** Shanna S. Leventhal, Carl Shaia, Deepashri Rao, Matthew Lewis, Kimberly Meade-White, Jesse H. Erasmus, Heinz Feldmann, David W. Hawman

**Affiliations:** 1 Laboratory of Virology, Division of Intramural Research, National Institute of Allergy and Infectious Diseases, National Institutes of Health, Rocky Mountain Laboratories, Hamilton, MT USA; 2 Rocky Mountain Veterinary Branch, Division of Intramural Research, National Institute of Allergy and Infectious Diseases, National Institutes of Health, Rocky Mountain Laboratories, Hamilton, MT USA; 3 HDT Bio, Seattle, WA USA

**Keywords:** RNA vaccines, Virology

## Abstract

Spread by *Hyalomma* genus ticks, Crimean-Congo hemorrhagic fever virus (CCHFV) causes a severe hemorrhagic disease endemic throughout Southern and Eastern Europe, Asia, and Africa. To date, there are no widely approved vaccines for CCHFV and treatment for disease is largely supportive. Due to this lack of intervention, the WHO lists CCHFV as a high-priority pathogen. Recently, we described a highly efficacious self-replicating RNA vaccine which is protective against CCHFV disease in mice and non-human primates. This vaccine induces high titers of non-neutralizing anti-nucleoprotein (NP) antibodies and a robust T-cell response against the viral glycoprotein. Here, we assess the durability of this vaccine in mice by monitoring the immunogenicity and efficacy of this vaccine up to 1 year post vaccination. We found that while glycoprotein-specific T-cell responses and anti-NP antibody titers waned over time, mice remained protected against lethal CCHFV challenge for at least 1 year post vaccination.

## Introduction

Crimean Congo hemorrhagic fever virus (CCHFV) is the most prevalent tick-borne bunyavirus, being endemic to Europe, the Middle East, Asia, and Africa. Spread by the bite of *Hyalomma* genus ticks, the main reservoir of the virus, in humans, CCHFV causes a severe hemorrhagic disease with case fatality rates of 30% or higher^1^. Although CCHFV is not known to cause disease in other species, it does result in asymptomatic infection of livestock and small animal species^2^. Livestock serve as amplifying hosts of CCHFV, and human infection can occur when coming into contact with infected blood, such as during slaughter and butchering^1^. There have also been several reports of nosocomial infections spread primarily between patients and healthcare workers^3^. Although many cases in humans are asymptomatic, disease may begin as a nonspecific febrile illness with symptoms including headache, fever, and myalgia before progressing into a severe stage characterized by multi-systemic hemorrhage^1^. Notably, the World Health Organization lists CCHFV as a high-priority pathogen as there are no widely approved vaccines or therapeutics, and interventions are limited to prevention and supportive care.

CCHFV is a negative-sense, RNA bunyavirus which has a tri-segmented genome composed of the small (S), medium (M), and large (L) genomic segments^4^. The S segment encodes for the viral nucleoprotein (NP) as well as a small non-structural protein (NSs) while the M segment encodes a glycoprotein precursor (GPC) which is later cleaved into the glycoproteins (Gn and Gc) as well as several nonstructural proteins including the mucin-like domain, GP38, and nonstructural M segment protein^4^. The L segment encodes for the RNA-dependent RNA-polymerase and an OTU domain but has several regions of unknown function^1,4^. Both the NP and GPC have been evaluated as vaccine candidates; however, correlates of protection are unclear, and results have varied across antigens and vaccine platforms^1^. We developed an alphavirus-based replicating RNA vaccine which encodes the CCHFV NP (repNP) and CCHFV Gc glycoprotein (repGc) which is protective in both mice and non-human primates (NHPs) against CCHFV challenge^5–7^. While repNP induces a robust, non-neutralizing anti-NP antibody response, repGc induces a robust T-cell response in mice^5–7^. Due to CCHFV being endemic in regions with limited healthcare resources, an ideal vaccine would induce durable immunity without a need for continuous boosting. Here, we assess the durability of these protective immune responses in mice up to a year post-vaccination in a lethal CCHFV challenge model.

## Results

### Anti-NP antibody titers and Gc-specific T-cells persist for at least 1-year post-vaccination

**Fig. 1:**
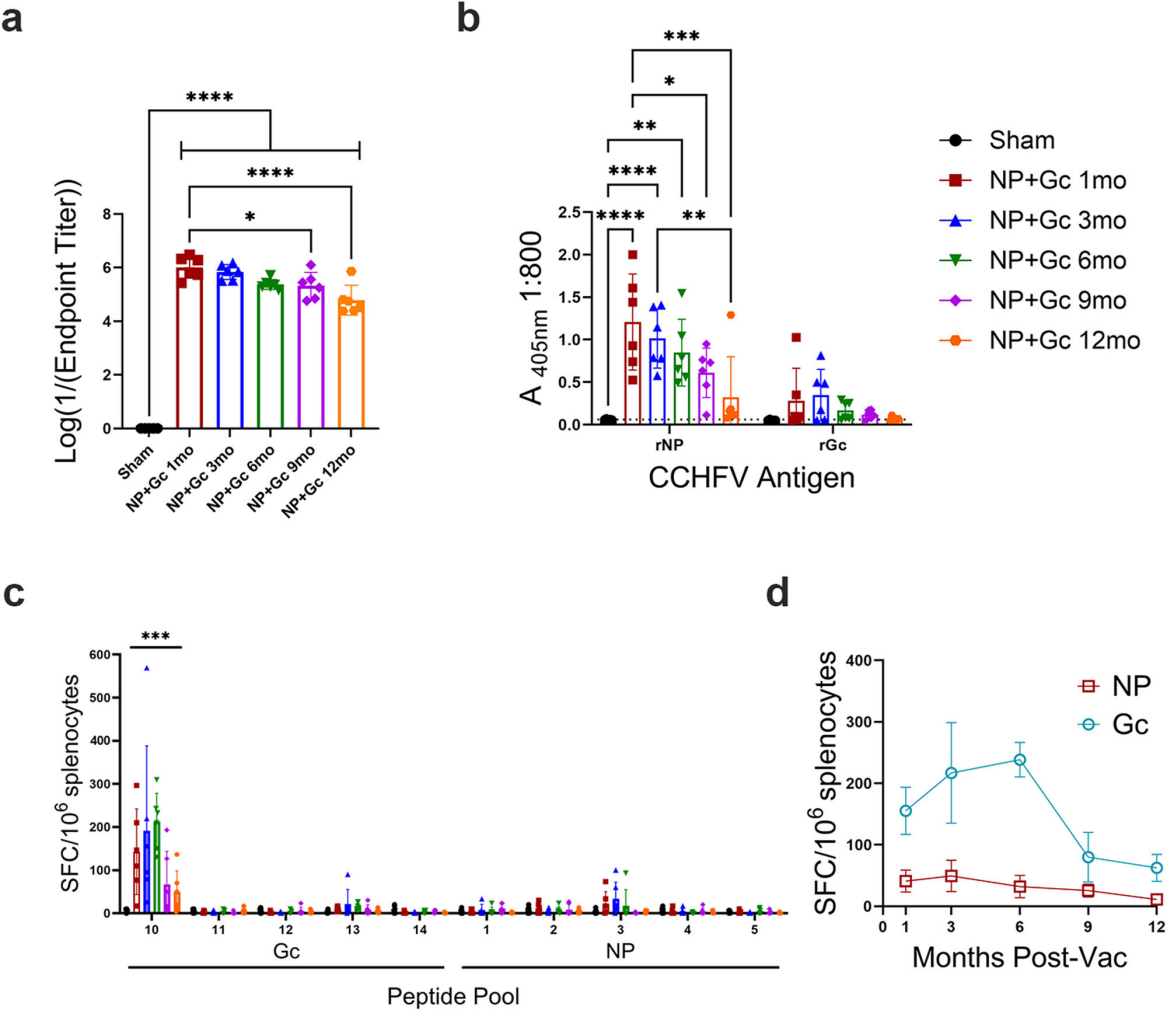
repNP + repGc vaccination elicits humoral and cellular immunity up to 1 year post-vaccination. WT C57BL6/J mice were vaccinated prime boost with 1 µg repNP + repGc RNA 4 weeks apart. Immunogenicity of vaccination was measured 1-, 3-, 6-, 9-, and 12-months post-boost vaccination. At each time point, groups of six mice were evaluated for CCHFV specific immune responses via **a** whole virion IgG ELISA shown with endpoint titers and **b** recombinant antigen ELISA to the CCHFV nucleocapsid (NP) and glycoprotein c (Gc) proteins. Sham mice are combined from all timepoints. Dashed lines indicate background absorbance of wells. CCHFV-specific T-cell responses were measured via IFNγ ELISpot shown as **c** cumulative spot-forming cells (SFCs) against peptide pools spanning the entire NP or Gc as well as **d** cumulative responses to NP and Gc overtime. Statistical comparisons were calculated using a one-way ANOVA with Turkey’s multiple comparison test. ns *P* > 0.05, *P < 0.05, **P < 0.01, ***P < 0.001, ****P < 0.0001. Data are shown as mean plus standard deviation.

To assess the durability of our repNP + repGc vaccination, we immunized mice with a prime-boost regimen, 4 weeks apart, with 1 µg total RNA at each immunization and evaluated immunogenicity at 1, 3-, 6-, 9-, and 12-months post-boost. As a control, each time point included mice vaccinated with a repRNA expressing the irrelevant green fluorescent protein (sham). As expected, 1-month after the boost, mice vaccinated with repNP + repGc had robust CCHVF-specific IgG antibody titers which were predominantly against the CCHFV NP (Fig. 1a, b). These titers peaked at 1-month post-boost (mean endpoint titer >1/755,000) and while they waned over time, were still detectable at 12 months post vaccination (mean endpoint titer >1/27,000) (Fig. 1a, b). Similar to early timepoints, at later timepoints antibodies remained predominantly against the CCHFV NP and responses against the CCHFV glycoprotein c (Gc) were minimal (Fig. 1b). As seen previously^7^, T-cell responses were primarily against the Gc protein peptide pool 10 (a.a. 1081–1211), which spans the N-terminal portion of Gc (Fig. 1c). CCHFV-specific T-cell responses peaked at 6-months post-vaccination (mean >230 SFCs/10^6^ splenocytes) and waned thereafter but were still significantly increased compared to sham vaccinated animals 12 months post-vaccination (mean >60 SFCs/10^6^ splenocytes) (Fig. 1d). NP-specific T-cell responses were minimal and did not significantly increase above sham vaccinated animals at any timepoint (Fig. 1c, d). Cumulatively, repNP + repGc vaccination induced CCHFV-specific antibody and T-cell responses which persisted up to 1-year post-vaccination and no sex-related differences were observed in immune responses for any group. Similar to shortly after vaccination, antibody responses were primarily against the CCHFV NP while T-cell responses were primarily against the CCHFV Gc.

### repNP + repGc vaccination confers significant protection for at least 1-year post-vaccination

**Fig. 2:**
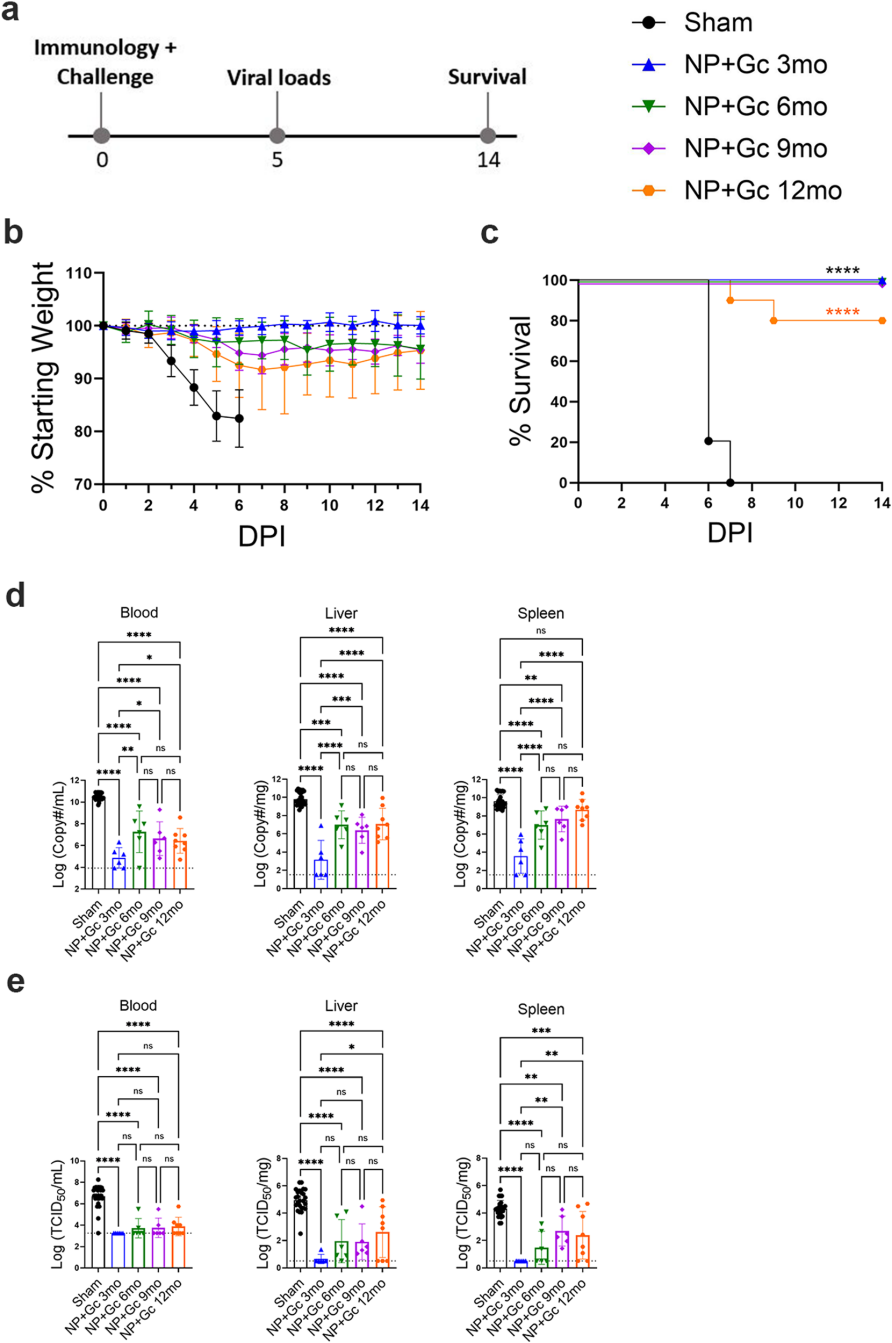
repNP + repGc vaccination is protective up to 1-year post-vaccination. Groups of WT C57BL6/J mice vaccinated prime-boost with repNP + repGc were treated with MAR1-5A3 antibody to block the type I IFN response and infected with 100 TCID_50_ CCHFV strain UG3010 3-, 6-, 9-, and 12-months post-boost vaccination. At each timepoint, mice were **a** challenged on “day 0” and groups of mice were assessed for control of viral loads and survival on days 5 and to day 14, respectively, relative to CCHFV challenge. Mice (*N* = 8) were monitored for **b** weight loss and **c** survival over the course of infection. On day 5 post-infection, mice (*N* = 6) were evaluated for control of **d** viral genome copies via qRT-PCR and **e** infectious virus via TCID_50_ in the blood, liver, and spleen. Dashed lines indicate limit of detection. Statistical comparisons were calculated using a one-way ANOVA with Turkey’s multiple comparison test. ns >0.05, *P < 0.05, **P < 0.01, ***P < 0.001, ****P < 0.0001. Data are shown as mean plus standard deviation.

We next evaluated the efficacy of immune responses over time by challenging groups of mice with a lethal, heterologous CCHFV challenge at 3-, 6-, 9-, and 12-months post-boost vaccination. Our challenge model utilizes C57BL6/J mice treated with the type I IFN suppressing antibody MAR1-5A3 and infected with 100 TCID_50_ CCHFV strain UG3010. Our vaccine antigens are based on CCHFV strain Hoti and this represents a stringent heterologous challenge model. At indicated timepoints post-boost, mice were challenged on “day 0” relative to CCHFV challenge and assessed for control of viral loads, weight loss, and survival over the course of study (Fig. 2a). Sham vaccinated mice from all timepoints consistently began to lose weight at 3 days post infection (d.p.i.) and succumbed to disease by day 7 (Fig. 2b, c), demonstrating that this lethal challenge model remains lethal even in mice over 1 year in age. Compared to sham vaccinated mice, repNP + repGc vaccinated mice were significantly protected from lethal disease for at least 1-year post-vaccination (Fig. 2c). At 3 months, we saw no signs of clinical disease (Fig. 2b), similar to what we have previously reported at 1-month post-vaccination. At later timepoints post-vaccination, mice experienced mild weight loss, beginning at around 5 d.p.i. (Fig. 2b) suggesting breakthrough infection. However, vaccination conferred 100% survival up to 9 months post-vaccination and 80% survival (8/10 mice survived) in the 12-month group (Fig. 2c). Together, these data indicate that our vaccine administered as a prime-boost confers durable protection against lethal CCHFV infection for at least 1-year after vaccination.

We also evaluated viral loads in the blood, liver, and spleen by qRT-PCR and infectious titration. Similar to 1-month post-vaccination^7^, at 3-months post-vaccination, viral RNA was significantly reduced and little to no infectious virus was detectable in these tissues (Fig. 2d, e). Interestingly, although mice were protected from lethal disease out to 12-months, breakthrough in viral control was detected beginning 6-months post-vaccination (Fig. 2d, e). Although viral genome copies were significantly lower in the blood, liver, and spleen than sham vaccinated animals at 6- and 9-months post-vaccination, they were also significantly higher compared to the 3-month group (Fig. 2d). At 12-months, viral RNA remained significantly lower than sham vaccinated animals in the blood and liver but not the spleen (Fig. 2d). However, infectious virus in the blood, liver, and spleen was significantly decreased compared to sham vaccinated animals at all timepoints (Fig. 2e) although, titers were significantly higher in the liver and spleen in the 9-and 12-month groups compared to the 3-month group (Fig. 2e). Cumulatively, our data shows that repNP + repGc vaccination confers durable protection against lethal CCHFV challenge in mice out to 1-year post vaccination.

### repNP + repGc vaccination protects mice from hepatic and splenic pathology 1-year post-vaccination

**Fig. 3:**
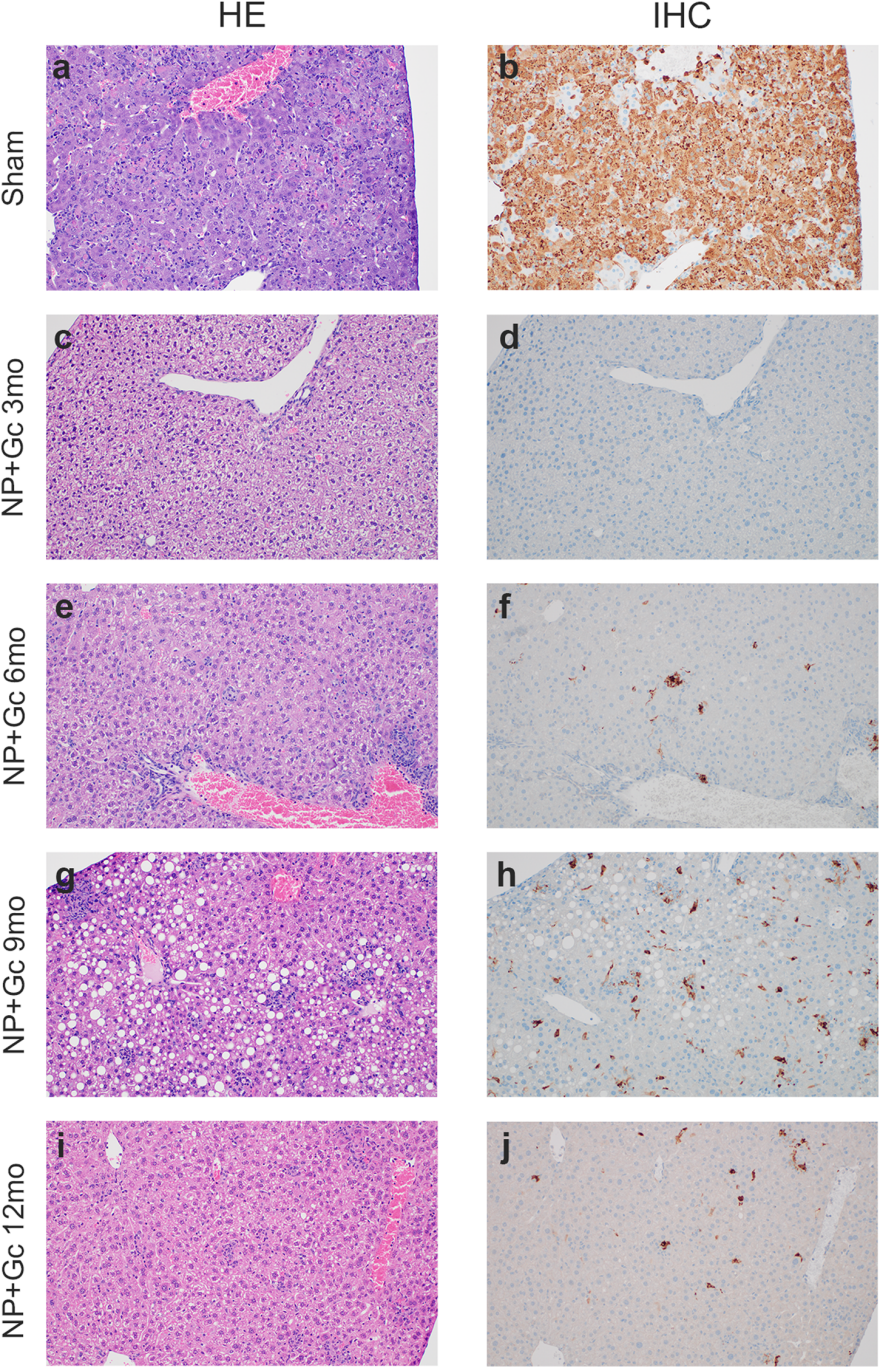
Histologic findings in the liver. Groups of WT mice sham vaccinated (**a**, **b**) or vaccinated with repNP + repGc and challenged at 3mo (**c**, **d**), 6mo (**e**, **f**), 9mo (**g**, **h**), or 12mo (**i**, **j**) post-vaccination were euthanized on day 5 p.i. and liver collected, and formalin fixed. Sections were (left) H&E stained or (right) probed for the presence of viral antigen via immunohistochemistry (IHC). Representative images for each group are shown at 200× magnification. Complete findings are provided in Supplementary Table 1.

To further assess the durability of repNP + repGc vaccination, we evaluated spleen and liver samples, key tissues of CCHFV pathogenesis, from infected mice 5 d.p.i, during peak disease. The sham-vaccinated mice from each group developed histologic lesions and immunoreactivity results consistent with CCHFV infection in mice (Fig. 3a, b, Supplementary Fig. 1a, b). Hepatic lesions consist of multifocal to diffuse hepatocellular degeneration and necrosis with neutrophilic and histiocytic inflammation (Fig. 3a). Anti-CCHFV immunohistochemistry in sham vaccinated mice reveals near diffuse hepatocellular immunoreactivity (Fig. 3b). Splenic lesions consisted of necrosis and loss of white pulp with swollen red pulp macrophages and varying amounts of necrotic debris (Supplementary Fig. 1a). Immunohistochemistry revealed a near diffuse immunoreactivity of red pulp macrophages and occasional white pulp mononuclear cells believed to be macrophages, lymphocytes or follicular dendritic cells or any combination of the three (Supplementary Fig. 1b). These data suggest that older mice, in this study up to 56 weeks of age at time of challenge, develop similar CCHFV-induced pathology compared to younger mice.

In contrast to sham-vaccinated mice, 3-months post vaccination repNP + repGc vaccinated mice had no evidence of pathology or CCHFV antigen in the liver or spleen (Fig. 3c, d and Supplementary Fig. 1c, d). At 6- and 9- months post-vaccination, and consistent with breakthrough infection seen in weight loss and control of viral replication, vaccinated mice developed minimal to moderate histologic lesions and immunoreactivity consistent with CCHFV infection in 5/6 mice (Fig. 3e–h, Supplementary Fig. 1e–h). At 12-months post vaccination all 8 repNP + repGc vaccinated mice developed mild histologic lesions and minimal to marked immunoreactivity consistent with CCHFV infection and similar to the 6- and 9- month groups (Fig. 3i, j and Supplementary Fig. 1i, j). Despite evidence of pathology and immunoreactivity in vaccinated mice at later time points post-vaccination, at all timepoints the histologic lesions and immunoreactivity of the vaccinated mice were less than that of the sham vaccinated control mice.

### Rapid anamnestic immune responses at 6-, 9-, and 12-months post vaccination

**Fig. 4:**
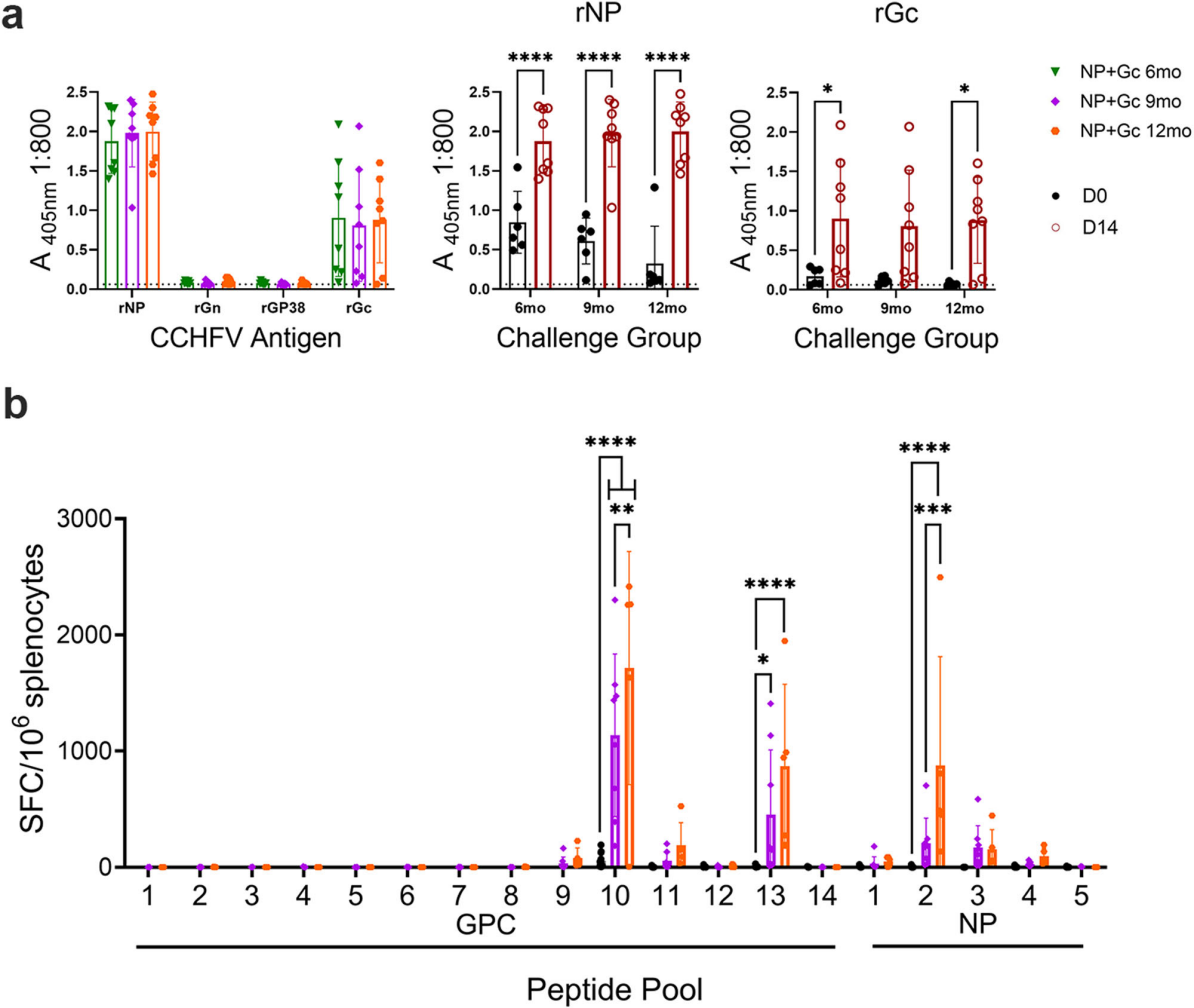
Anamnestic responses to CCHFV infection. Surviving mice from repNP + repGc 6-, 9-, and 12-months groups were evaluated on day 14 p.i. for CCHFV-specific anamnestic responses. Sera was collected and evaluated for CCHFV-specific antibodies using **a** recombinant antigen ELISA to the CCHFV NP, Gn, GP38, and Gc. Responses are shown for the 6-, 9-, and 12-month groups as well as comparisons between D0 and D14 for CCHFV-NP and Gc responses. Further, anamnestic CCHFV-specific T-cell responses were measured via **b** IFNγ ELISpot shown as cumulative SFCs against peptide pools spanning the entire GPC or NP as well as a comparison between D0 and D15 responses to the GPC peptide pool 10. Data from day 0 is duplicated from Fig. 1 and shown here for comparison. Dashed lines indicate limit of detection. Statistical comparisons were calculated using a one-way ANOVA with Turkey’s multiple comparison test. ns >0.05, *P < 0.05, **P < 0.01, ***P < 0.001, ****P < 0.0001. Data are shown as mean plus standard deviation.

Previously, we showed that prime-boost vaccination with repNP + repGc was protective against lethal CCHFV challenge in mice one month post vaccination and surviving mice from these studies did not develop CCHFV-specific anamnestic responses^7^. However, since we observed breakthrough viral replication at 6-months post-vaccination, yet animals remained significantly protected against CCHFV disease, we hypothesized that rapid anamnestic humoral or cellular immunity may contribute to protection from CCHFV challenge at later timepoints post-vaccination. In surviving mice challenged at 6-, 9- and 12-months post-vaccination, we evaluated antibody responses using recombinant antigens, including Gn and GP38 antigens that were not present in the vaccine. We also measured T-cell responses in surviving vaccinated mice challenged at 9- and 12-months post-vaccination with peptides spanning the NP and entire GPC, including regions not present in the repGc vaccine. Two-weeks after CCHFV challenge, surviving mice challenged at 6-, 9-, and 12-months post-vaccination, had increases in both anti-NP and anti-Gc antibody titers compared to day 0 (Fig. 4a), consistent with breakthrough viral replication and transient clinical disease (Figs. 2b–e and 4a). Interestingly, although we failed to detect antibodies against the Gc prior to infection, after infection, surviving mice had increased antibody response to the Gc that was significant at 6- and 12-month post-vaccination (Fig. 4a). Previously we have shown that anti-Gc anamnestic antibody responses post-repNP and repGc vaccination and CCHFV challenge do not result in neutralizing activity^7^. No group had a response to the CCHFV Gn or GP38 proteins (Fig. 4a). In addition, anamnestic T-cell responses were observed against the GPC peptide pool 10, similar to the specificity of T cells prior to challenge, while mice also developed significant responses against GPC pool 13 and NP peptide pool 2 (Fig. 4b) suggesting a broadening of the CCHFV-specific T-cell response after infection. Together, these data support a hypothesis that repNP + repGc vaccination confers durable immunity against CCHFV challenge through both preexisting and rapid recall responses upon viral challenge.

## Discussion

With the increasing geographic range of CCHFV putting more populations at risk of infection, there is a significant public health need for a vaccine. However, there are no widely approved vaccines and only an inactivated CCHFV vaccine is commonly used in Bulgaria^1,8^. However, due to safety concerns, this vaccine is unlikely to achieve widespread usage^1^. Here, we report the durability of a repRNA vaccine for CCHFV expressing the viral NP or Gc glycoprotein. These findings add to our previous results demonstrating that this vaccine confers rapid protection against lethal challenge in mice in as little as 2-weeks and confers protection against disease in an NHP model^6,7,9^. Here we demonstrate that protection is maintained for at least a year post-vaccination.

To date, there is no consensus on what antigens and immune responses are required for protection by CCHFV vaccines and the durability of these protective responses is similarly poorly understood. In one study, inactivated vaccines derived from cell culture (CCVax) and mouse brain (MBVax) were compared over the course of a year at 5, 10, and 20 µg doses administered as a prime-boost-boost in mice^10^. Similar to our vaccine, antibody titers peaked 4 months post-vaccination and then decreased over time^10^. Interestingly, these vaccines developed neutralizing antibodies^10^ while our vaccine did not induce significant antibody responses to the Gc. However, CCVax and MBVax were not evaluated for efficacy against CCHFV challenge. In other vaccine platforms, neutralizing antibodies are not always required for protection. In one study, a nucleoside-modified mRNA lipid nanoparticle encoding the CCHFV GnGc induced a robust T-cell response and neutralizing antibodies which protected mice from lethal CCHFV challenge^11^. However, other vaccine platforms expressing the Gn and Gc, including a subunit and DNA vaccine, induced neutralizing antibodies which were not protective or partially protective, respectively^12,13^. Further, in passive immunization experiments, some neutralizing anti-Gc antibodies were not protective while non-neutralizing anti-Gn and anti-GP38 antibodies could confer complete protection^14,15^. Lastly, the Bulgarian vaccine has not induced high titers of neutralizing antibodies even after four doses^16^, further indicating that neutralizing antibodies are not essential for protection.

In our previous studies on repNP and repGc vaccinations, we found that protection was primarily conferred by non-neutralizing, anti-NP antibodies and that diminished antibody titers correlated with decreased protection^6,7^. Here, we observed anti-NP antibody titers waning over time and this correlated with incomplete protection at the 12-month post-vaccination challenge. These findings continue to support the role of anti-NP antibodies in vaccine-mediated protection and ongoing work has identified a requirement of the intracellular Fc-receptor tripartite motif-containing protein 21 (TRIM21) in mediating this protection^17^. Although we have shown repGc T-cell responses to be partially protective^6,7^, these responses also significantly waned over a 12-month period. While CCVax and MBVax did not assess T-cell responses over time, the Bulgarian vaccine is known to induce a robust T-cell response although it requires boosting every 5 years and its efficacy has not been formally assessed^8,10,16^. A modified *vaccinia* virus and DNA vaccines expressing the CCHFV glycoproteins also induced robust T-cell and antibody responses and protected mice against lethal CCHFV challenge, indicating that in other platforms T cells may play an essential role in protection^18–20^.

Consistent with a decrease in control of viral replication, we observed significant anamnestic immune responses at 6-, 9-, and 12-months post vaccination. This included significant increases in anti-NP and anti-Gc antibody responses and an increase in CCHFV-Gc and NP-specific T cells (Fig. 4). Interestingly, although there were no significant differences in viral genome copies or infectious virus in the blood, liver, and spleen between the 6- and 9-month groups and the 12-month group, we observed breakthrough clinical disease and lethality in only the 12-month group (Fig. 2). These data suggest that although antibody titers waned over time, they remained above levels necessary to prevent lethal disease in all mice for at least 9 months and in most mice for at least 12 months post-vaccination. While anamnestic antibody titers against the NP and Gc were consistent between the 6-, 9- and 12-month groups, there was a significant increase in NP-specific T-cell responses only in the 12-month group and we have not previously observed a strong NP T-cell response from this vaccine^6,7^. It is unclear if the T-cell responses directed against the NP measured after challenge represent long-term recall responses or de novo responses. In studies in humans vaccinated with the Bulgarian vaccine, robust T-cell responses were observed against the NP, and NP-specific CD8 + T cells were found in healthy individuals’ years after infection^16,21^. Two CCHFV vaccines using solely the NP have observed NP-specific T-cell responses but contribution to protection was not assessed^22,23^. To date, it is unclear if NP-specific T-cell responses contribute to protection although we have previously shown that our vaccine remains effective in mice depleted of T cells^6^.

Our study has some important limitations. First, we have recently shown that in NHPs a repRNA expressing the full-length GPC was largely non-immunogenic and likely did not contribute to protection observed in that model^5^. Thus, the durable T-cell responses elicited by the repGc vaccine here may not be seen in NHPs or humans. However, extensive mouse and NHP data indicate that NP-specific antibodies are sufficient for protection and our data here demonstrate that these NP-specific antibodies remain above protective levels for at least 12-months post-vaccination. Second, we have previously shown that our vaccine could protect after a single immunization while here we measured the durability of protection in a prime-boost regimen. While we have previously shown that boosting in mice failed to increase short-term immune responses to the vaccine^6^, the durability of immune responses after a single immunization will require further study. Lastly, while we have demonstrated durable protection for at least a year post-vaccination, we measured an ongoing trend of waning immunity suggesting that eventually vaccine-elicited immunity may decline below protective levels. This suggests that continued booster vaccinations may be needed. However, it is possible that this trend is due to the increasing age, shorter lifespan, and decline of the mouse immune system^24–26^. Nonetheless, given the stringent challenge and rapid onset of lethal disease in our immunocompromised mouse challenge model, these long-term memory responses may be even more effective at controlling infection in immunocompetent hosts. Further, studies of human CCHF survivors suggest that immunity following CCHFV infection may last for years, possibly lifelong^21,27^ and no known cases of reinfection with CCHFV have been reported. In addition, our study began with mice vaccinated at 8 weeks of age and mice were challenged at ages corresponding to human “mature adult” (5 months of age) and “middle-aged” (8, 11, and 14 months of age), further indicating that repRNA induced immunity may be durable^26^. Thus, multi-year protective immunity to CCHFV may be possible and further studies, particularly in NHPs or human clinical trials, will be needed to define the length of protection afforded by the repRNA vaccine.

In summary, we have shown that our repRNA vaccine elicits long-lived humoral and cellular immune responses that significantly protect against lethal, heterologous CCHFV challenge in mice for at least 1 year after the last vaccination. Our data significantly increase our understanding of the kinetics of repRNA vaccine-elicited antibodies and T-cell responses and indicate that this vaccine primes a long-term memory response that can rapidly respond to CCHFV infection. Together with our previous reports showing rapid, single-shot protection against CCHFV infection, our findings support the continued development of our vaccine in clinical trials.

## Methods

### Ethics

All animal work was approved by the Rocky Mountain Laboratories Institutional Animal Care and Use Committee (protocol #2020-010) in accordance with recommendations by the Guide for the Care and Use of Laboratory Animals of the National Institutes of Health, the Office of Animal Welfare, the United States Department of Agriculture in an association for Assessment and Accreditation of Laboratory Animal Care-Accredited Facility. Mice were housed in HEPA-filter cage systems enriched with nesting material and commercial food and water available ad libitum. All work with infectious CCHFV was done following guidelines put forth by the Institutional Biosafety Committee in biocontainment level 4 at the Rocky Mountain Laboratories, NIAID, NIH, Hamilton, MT.

### Mice, vaccinations, and infections

Male and female wild-type C57BL6/J mice (stock #00664) were purchased from Jackson Laboratories. Mice were assigned randomly to a group and all groups consisted of equal numbers of male and female mice. The vaccine was derived from CCHFV strain Hoti and prepared as previously described^28^. Mice were vaccinated prime-boost 4 weeks apart starting at ~8-weeks of age via a single 50 µL intramuscular injection delivering a total of 1 µg RNA to the hind limb. Vaccination appeared well tolerated and no adverse events attributable to vaccination were observed. At time of challenge, mice were treated with 2.5 mg MAR1-5A3 antibody (Leinco) via a single intraperitoneal injection followed by infection with a lethal dose of 100 TCID_50_ CCHFV strain UG3010 via single intraperitoneal injection. MAR1-5A3 suppresses the type I immune response, rendering mice immunosuppressed and susceptible to CCHFV at the time of challenge. All procedures (vaccinations, infections, treatments) were done under deep anesthesia by isoflurane inhalation. Euthanasia was performed by trained personnel using exsanguination under deep anesthesia followed by cervical dislocation or isoflurane overdose followed by cervical dislocation. CCHFV strain UG3010 was originally provided by Eric Bergeron, Centers for Disease Control and Prevention, and was grown, titrated, and verified onsite as previously described^6^.

### Enzyme-linked immunosorbent assay (ELISA)

In-house ELISAs using whole CCHFV antigen or recombinant antigen (Native Antigen) were used to quantify CCHFV-specific antibody response as previously described^7,29^. Briefly, antigen was adsorbed to NUNC high-binding plates overnight followed by incubation with mouse sera, secondary antibody, and ABTS development solution. Absorbance was read using Biotek plate reader. For recombinant antigen ELISAs, recombinant rNP, rGn, rGP38, or rGc (Native Antigen) was adsorbed to plates.

### Enzyme-linked immunosorbent spot assay (ELISpot)

CCHFV-specific T-cell responses were evaluated as previously described using CCHFV strain Hoti peptides (Genscript) and mouse single-color IFNγ kit (ImmunoSpot)^6^.

### Quantitative reverse-transcription PCR (qRT-PCR)

RNA was extracted from blood and tissue samples using Qiagen Qiamp viral RNA-mini-isolation kit and Qiagen RNeasy mini-isolation kit, respectively, and provided protocols. Viral RNA was quantified as previously described^6^.

### Median tissue culture infectious dose assay (TCID_50_)

Infectious virus in blood and tissue samples was quantified as previously described^6^. Briefly, blood and homogenized tissue samples were diluted and plated over SW13 cells and incubated over 5 days to observe any cytopathic effects. TCID_50_ was calculated using the Reed & Muench method^30^.

### Histology and immunohistochemistry

Tissues were fixed in 10% Neutral Buffered Formalin ×2 changes, for a minimum of 7 days. Tissues were placed in cassettes and processed with a Sakura VIP-6 Tissue Tek, on a 12-h automated schedule, using a graded series of ethanol, xylene, and PureAffin. Embedded tissues were sectioned at 5 μm and dried overnight at 42 °C prior to staining. Specific anti-CCHFV immunoreactivity was detected using Rabbit anti-CCHFV N IBT (Bioservices, cat#04-0011) at a 1:2000 dilution. The secondary antibody is the Immpress-VR horse anti-rabbit IgG polymer kit Vector Laboratories cat#MP-6401. The tissues were then processed for immunohistochemistry using the Discovery Ultra automated stainer (Ventana Medical Systems) with a ChromoMap DAB kit Roche Tissue Diagnostics cat#760-159.

### Statistics

Indicated statistical tests were performed using Graph-Pad Prism 10. Sample size was determined from our experience with CCHFV mouse models and animals were randomly assigned to study groups and time points. Pathologists were blinded to study groups.

## Supplementary information

Supplementary Information

## Data Availability

All data presented are available upon request.

## References

[1] Hawman, D. W. & Feldmann, H. Crimean-Congo haemorrhagic fever virus. *Nat. Rev. Microbiol.* **21**, 463–477 (2023). 36918725 10.1038/s41579-023-00871-9 PMC10013989

[2] Spengler, J. R., Bergeron, E. & Rollin, P. E. Seroepidemiological studies of crimean-congo hemorrhagic fever virus in domestic and wild animals. *PLoS Negl. Trop. Dis.* **10**, e0004210 (2016). 26741652 10.1371/journal.pntd.0004210 PMC4704823

[3] Tsergouli, K. et al. Nosocomial infections caused by Crimean-Congo haemorrhagic fever virus. *J. Hosp. Infect.* **105**, 43–52 (2020). 31821852 10.1016/j.jhin.2019.12.001

[4] Leventhal, S. S. et al. A look into *Bunyavirales Genomes*: functions of non-structural (NS) proteins. *Viruses* **13**, 314 (2021). 33670641 10.3390/v13020314 PMC7922539

[5] Hawman, D. W. et al. A replicating RNA vaccine confers protection in a rhesus macaque model of Crimean-Congo hemorrhagic fever. *NPJ Vaccines* **9**, 86 (2024). 38769294 10.1038/s41541-024-00887-z PMC11106275

[6] Leventhal, S. S. et al. Replicating RNA vaccination elicits an unexpected immune response that efficiently protects mice against lethal Crimean-Congo hemorrhagic fever virus challenge. *EBioMedicine* **82**, 104188 (2022). 35907368 10.1016/j.ebiom.2022.104188 PMC9335360

[7] Leventhal, S. S. et al. Single dose, dual antigen RNA vaccines protect against lethal Crimean-Congo haemorrhagic fever virus infection in mice. *EBioMedicine* **101**, 105017 (2024). 38382314 10.1016/j.ebiom.2024.105017 PMC10885550

[8] Ahata, B. & Akcapinar, G. B. CCHFV vaccine development, current challenges, limitations, and future directions. *Front. Immunol.* **14**, 1238882 (2023). 37753088 10.3389/fimmu.2023.1238882 PMC10518622

[9] Hawman, D. et al. A replicating RNA vaccine confers protection in a rhesus macaque model of Crimean-Congo hemorrhagic fever. *npj Vaccines* **9***,* 86 (2024). 10.1038/s41541-024-00887-z PMC11106275 38769294

[10] Berber, E. et al. Development of a protective inactivated vaccine against Crimean-Congo hemorrhagic fever infection. *Heliyon* **7**, e08161 (2021). 34703927 10.1016/j.heliyon.2021.e08161 PMC8526982

[11] Appelberg, S. et al. Nucleoside-modified mRNA vaccines protect IFNAR(-/-) mice against Crimean Congo hemorrhagic fever virus infection. *J. Virol.* **96***,* e0156821 (2021). 10.1128/jvi.01568-21 PMC8826901 34817199

[12] Garrison, A. R. et al. A DNA vaccine for Crimean-Congo hemorrhagic fever protects against disease and death in two lethal mouse models. *PLoS Negl. Trop. Dis.* **11**, e0005908 (2017). 28922426 10.1371/journal.pntd.0005908 PMC5619839

[13] Kortekaas, J. et al. Crimean-Congo hemorrhagic fever virus subunit vaccines induce high levels of neutralizing antibodies but no protection in STAT1 knockout mice. *Vector Borne Zoonotic Dis.* **15**, 759–764 (2015). 26684523 10.1089/vbz.2015.1855 PMC7643766

[14] Bertolotti-Ciarlet, A. et al. Cellular localization and antigenic characterization of crimean-congo hemorrhagic fever virus glycoproteins. *J. Virol.* **79**, 6152–6161 (2005). 15858000 10.1128/JVI.79.10.6152-6161.2005 PMC1091677

[15] Golden, J. W. et al. GP38-targeting monoclonal antibodies protect adult mice against lethal Crimean-Congo hemorrhagic fever virus infection. *Sci. Adv.* **5**, eaaw9535 (2019). 31309159 10.1126/sciadv.aaw9535 PMC6620094

[16] Mousavi-Jazi, M. et al. Healthy individuals’ immune response to the Bulgarian Crimean-Congo hemorrhagic fever virus vaccine. *Vaccine* **30**, 6225–6229 (2012). 22902680 10.1016/j.vaccine.2012.08.003

[17] Leventhal, S. S. et al. Antibodies targeting the Crimean-Congo hemorrhagic fever virus nucleoprotein protect via TRIM21. *Nat. Commun.* **15**, 9236 (2024). 39455551 10.1038/s41467-024-53362-7 PMC11511847

[18] Buttigieg, K. R. et al. A novel vaccine against Crimean-Congo haemorrhagic fever protects 100% of animals against lethal challenge in a mouse model. *PLoS ONE* **9**, e91516 (2014). 24621656 10.1371/journal.pone.0091516 PMC3951450

[19] Hinkula, J. et al. Immunization with DNA plasmids coding for Crimean-Congo hemorrhagic fever virus capsid and envelope proteins and/or virus-like particles induces protection and survival in challenged mice. *J. Virol.* **91**, e02076–16 (2017). 28250124 10.1128/JVI.02076-16 PMC5411611

[20] Golden, J. W. et al. Induced protection from a CCHFV-M DNA vaccine requires CD8(+) T cells. *Virus Res.* **334**, 199173 (2023). 37459918 10.1016/j.virusres.2023.199173 PMC10388194

[21] Goedhals, D., Paweska, J. T. & Burt, F. J. Long-lived CD8+ T cell responses following Crimean-Congo haemorrhagic fever virus infection. *PLoS Negl. Trop. Dis.* **11**, e0006149 (2017). 29261651 10.1371/journal.pntd.0006149 PMC5752039

[22] Dowall, S. D. et al. A Crimean-Congo hemorrhagic fever (CCHF) viral vaccine expressing nucleoprotein is immunogenic but fails to confer protection against lethal disease. *Hum. Vaccine Immunother.* **12**, 519–527 (2016). 10.1080/21645515.2015.1078045 PMC5049717 26309231

[23] Scholte, F. E. M. et al. Vaccination with the Crimean-Congo hemorrhagic fever virus viral replicon vaccine induces NP-based T-cell activation and antibodies possessing Fc-mediated effector functions. *Front Cell Infect. Microbiol.* **13**, 1233148 (2023). 37671145 10.3389/fcimb.2023.1233148 PMC10475602

[24] Xie, J. et al. The influences of age on T lymphocyte subsets in C57BL/6 mice. *Saudi J. Biol. Sci.* **24**, 108–113 (2017). 28053579 10.1016/j.sjbs.2016.09.002 PMC5198989

[25] Dutta, S. & Sengupta, P. Men and mice: relating their ages. *Life Sci.* **152**, 244–248 (2016). 26596563 10.1016/j.lfs.2015.10.025

[26] Hagan, C. When are mice considered old? [cited 2024]; Available from: https://www.jax.org/news-and-insights/jax-blog/2017/november/when-are-mice-considered-old (2017).

[27] Vasmehjani, A. A. et al. Persistence of IgG and neutralizing antibodies in Crimean-Congo hemorrhagic fever survivors. *J. Med. Virol.* **96**, e29581 (2024). 38572939 10.1002/jmv.29581

[28] Erasmus, J. H. et al. An *Alphavirus*-derived replicon RNA vaccine induces SARS-CoV-2 neutralizing antibody and T cell responses in mice and nonhuman primates. *Sci. Transl. Med.* **12**, eabc9396 (2020). 32690628 10.1126/scitranslmed.abc9396 PMC7402629

[29] Haddock, E. et al. A cynomolgus macaque model for Crimean-Congo haemorrhagic fever. *Nat. Microbiol.* **3**, 556–562 (2018). 29632370 10.1038/s41564-018-0141-7 PMC6717652

[30] Reed, L. J. & Muench, H. A simple method of estimating fifty percent endpoints12. *Am. J. Epidemiol.* **27**, 493–497 (1938).

