# Supplementary material for: Replicating RNA vaccine confers durable immunity against Crimean Congo hemorrhagic fever virus challenge in mice
Source: NPJ Vaccines. 2024 Dec 19;9:249. doi: 10.1038/s41541-024-01045-1 (PMC11659298; doi:10.1038/s41541-024-01045-1)
Supplement: Supplementary file 1 — Supplementary Information [file 41541_2024_1045_MOESM1_ESM.pdf]

Supplementary Figure 1

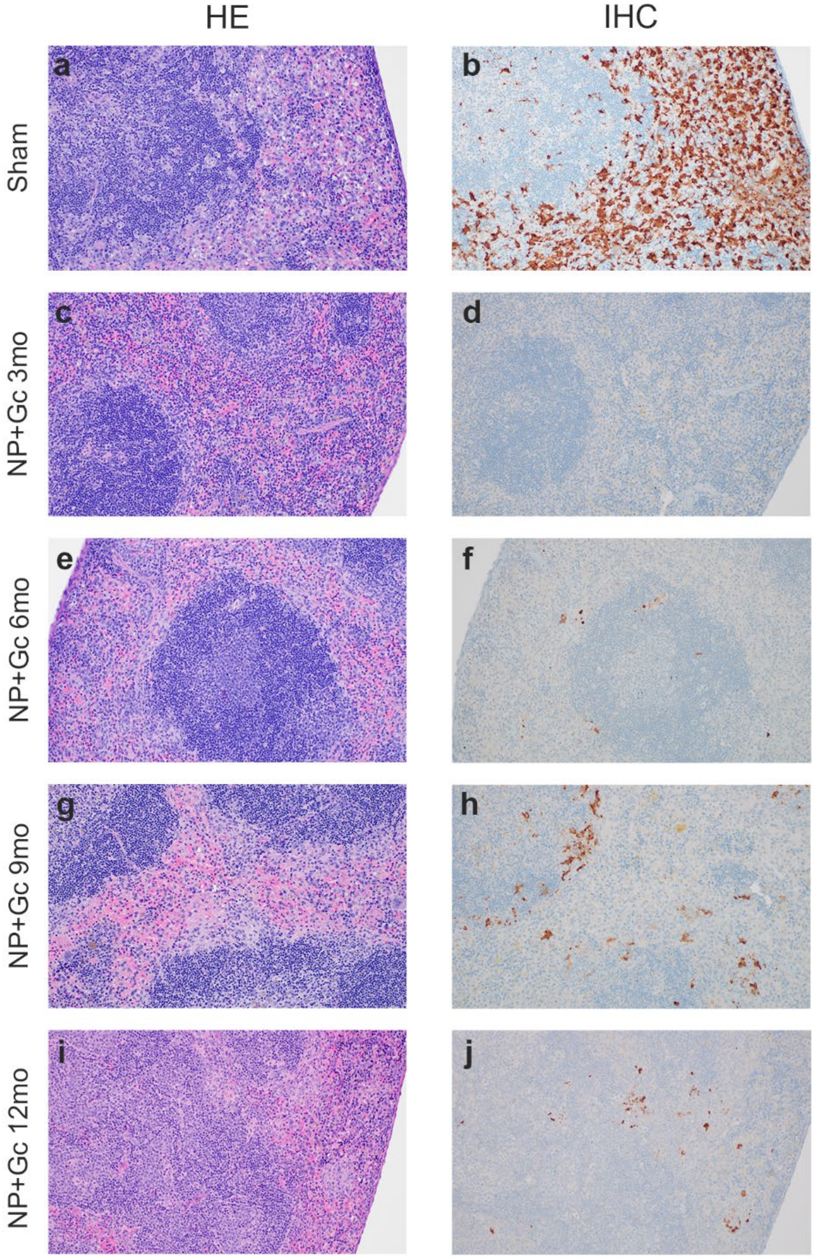

Supplementary Figure 1. Histological findings in the spleen. Groups of WT mice vaccinated with repNP + repGc and challenged at the indicated time points were euthanized on day 5 p.i. and spleen collected, and formalin fixed. Sections were (left) H&E stained or (right) probed for presence of viral antigen via immunohistochemistry (IHC). Representative images for each group are shown at 200X magnification. Complete findings are provided in Supplemental Table 1.

3 mos time point	M#53	M#54	M#55	M#56	M#57	M#58	M#59	M#60	M#61	M#62	M#63	M#64
group	Sham						NP+Gc					
sex	M	M	M	F	F	F	M	M	M	F	F	F
**Liver**												
inflammation, degeneration, necrosis	4	4	4	4	4	4	0	1	0	0	0	0
CCHF IHC	5	4	5	4	4	4	0	1	1b	0	0	0
**Spleen**												
increased tingible body macrophages	y	y	y	y	y	y	n	n	n	n	n	n
red pulp necrotic debris	y	y	y	y	y	y	n	n	n	n	n	n
white pulp necrosis/apoptosis/loss	y	y	y	y	y	y	n	n	n	n	n	n
pigment (p)												
CCHF IHC (red pulp mostly macrophages)	5	5	5	5	5	5	0	1b	1b	0	0	0
**6 mos time point**	M#93	M#94	M#95	M#96	M#97	M#98	M#99	M#100	M#101	M#102	M#103	M#104
group	Sham						NP+Gc					
sex	M died	M	M	F	F	F	M	M	M	F	F	F
**Liver**												
inflammation, degeneration, necrosis	4	4	4	4	4	4	2	1	3	0	2	3
CCHF IHC	4	4	4	4	4	4	2	1	3	0	1	3
**Spleen**												
increased tingible body macrophages	y	y	y	y	y	y	n	n	n	n	n	n
red pulp necrotic debris	y	y	y	y	y	y	n	n	y	n	n	y
white pulp necrosis/apoptosis/loss	y	y	y	y	y	y	n	n	n	n	n	n
pigment (p)												
CCHF IHC (red pulp mostly macrophages)	5	5	5	5	5	5	1	1b	2	0	0	2

9 mos time point	M#133	M#134	M#135	M#136	M#137	M#138	M#139	M#140	M#141	M#142	M#143	M#144

group	Sham						NP+Gc					
sex	M	M	M	F	F	F	M	M	M	F	F	F
**Liver**									nsf			
inflammation, degeneration, necrosis	4	4	4	4	4	4	3	3		2	2	3
CCHF IHC	5	5	5	4	4	4	3	1b	0	1b	2	3
**Spleen**												
increased tingible body macrophages	y	y	y	y	y	y	n	n	n	n	n	n
red pulp necrotic debris	y	y	y	y	y	y	y	n	n	n	n	n
white pulp necrosis/apoptosis/loss	y	y	y	y	y	y	y	n	n	n	n	n
pigment (p)										y	y	y
CCHF IHC (red pulp mostly macrophages)	5	5	5	5	5	5	3	1b	0	1b	1	2

**12 mos time point**	M#173	M#174	M#175	M#176	M#177	M#178	M#179	M#181	M#182	M#183	M#184	M#185	M#186	M#187	M#188
group	Sham							NP+Gc							
sex	M	M	M	M	M	F	F	M	M	M	M	M	F	F	F
**Liver**															
inflammation, degeneration, necrosis	4	4	4	3	4	3	4	2	2	2	2	2	2	2	2
CCHF IHC	4	4	5	4	5	4	4	2	3	2	2	3	1b	1	1
**Spleen**															
increased tingible body macrophages	y	y	y	y	y	y	y	n	n	n	n	n	n	n	n
red pulp necrotic debris	y	y	y	y	y	y	y	n	y	n	n	n	n	n	n
white pulp necrosis/apoptosis/loss	n	y	y	y	y	y	y	n	n	n	n	n	n	n	n
pigment (p)															
CCHF IHC (red pulp mostly macrophages)	5	5	5	5	5	5	5	2	4	2	2	3	1b	1b	1b

qns=quantity not sufficient	h=hemosiderin
nsf=no significant findings	g=glomeruli
n/a=not applicable	m=medulla
rp=red pulp	fh=follicular hyperplasia
wp=white pulp	1barely means I had to look around for a positive cell
b=both red and white pulp	y=yes; n = no

percent affected and distribution
0=None
1=1=10% (focal)
2=11-25% (multifocal)
3=26-50% (multifocal to coalescing)
4= 51-75% multifocal to coalescing)
5=76-100% (diffuse)

Supplementary Table 1. Complete histological Findings. Groups of WT mice vaccinated with repNP + repGc and challenged at the indicated time points were euthanized on day 5 p.i. and liver and spleen collected, and formalin fixed. Each group was analyzed and findings for each mouse are reported. M# - mouse number. 1b – 1 barely.
